# Increased breakdown of kynurenine towards its neurotoxic branch in bipolar disorder

**DOI:** 10.1371/journal.pone.0172699

**Published:** 2017-02-27

**Authors:** Armin Birner, Martina Platzer, Susanne Astrid Bengesser, Nina Dalkner, Frederike T. Fellendorf, Robert Queissner, Rene Pilz, Philipp Rauch, Alexander Maget, Carlo Hamm, Simone Herzog-Eberhard, Harald Mangge, Dietmar Fuchs, Natalie Moll, Sieglinde Zelzer, Gregor Schütze, Markus Schwarz, Bernd Reininghaus, Hans-Peter Kapfhammer, Eva Z. Reininghaus

**Affiliations:** 1 Department for Psychiatry and Psychotherapy, Medical University of Graz, Graz, Austria; 2 Clinical Institute of Medical and Chemical Laboratory Diagnostics, Medical University of Graz, Graz, Austria; 3 Division of Biological Chemistry, Biocenter, Medical University of Innsbruck, Innsbruck, Austria; 4 Institute of Laboratory Medicine, Medical Center of Munich University (LMU), Munich, Germany; University of Colorado Denver School of Medicine, UNITED STATES

## Abstract

**Introduction:**

Bipolar disorder (BD) is a chronic psychiatric disease which can take most different and unpredictable courses. It is accompanied by unspecific brainstructural changes and cognitive decline. The neurobiological underpinnings of these processes are still unclear. Emerging evidence suggests that tryptophan catabolites (TRYCATs), which involve all metabolites of tryptophan towards the kynurenine (KYN) branch, are involved in the etiology as well as in the course of BD. They are proposed to be mediators of immune-inflammation and neurodegeneration. In this study we measured the levels of KYN and its main catabolites consisting of the neurotoxic hydroxykynurenine (3-HK), the more neuroprotective kynurenic acid (KYNA) and anthranilic acid (AA) and evaluated the ratios between end-products and substrates as proxies for the specific enzymatic activity (3-HK/KYN, KYNA/KYN, AA/KYN) as well as 3-HK/KYNA as a proxy for neurotoxic vs. neuroprotective end-product relation in individuals with BD compared to healthy controls (HC).

**Methods:**

We took peripheral TRYCAT blood levels of 143 euthymic to mild depressive BD patients and 101 HC. For statistical analyses MANCOVA’s controlled for age, sex, body mass index, cardiovascular disease and smoking were performed.

**Results:**

The levels of KYNA (*F = 5*,*579; p <*.*05*) were reduced in BD compared to HC. The enzymatic activity of the kynurenine-3-monooxygenase (KMO) reflected by the 3-HK/KYN ratio was increased in BD individuals compared to HC (*F = 5*,*394; p <*.*05*). Additionally the ratio of 3-HK/KYNA was increased in individuals with BD compared to healthy controls (*F = 11*,*357; p <*.*01*).

**Discussion:**

In conclusion our findings subserve the concept of KYN -pathway alterations in the pathophysiology of BD. We present evidence of increased breakdown towards the neurotoxic branch in KYN metabolism even in a euthymic to mild depressive state in BD. From literature we know that depression and mania are accompanied by inflammatory states which should be capable to produce an even greater imbalance due to activation of key enzymes in the neurotoxic direction of KYN -conversion. These processes could finally be involved in the development of unspecific brain structural changes and cognitive deficits which are prevalent in BD. Further research should focus on state dependent changes in TRYCATs and its relation to cognition, brain structure and staging parameters.

## Introduction

Bipolar disorder (BD) is a chronic psychiatric disease which can take most different and unpredictable courses. It is accompanied by unspecific brain structural changes and cognitive decline [1–6]. In the absence of reliable biomarkers, the diagnosis and evaluation of treatment success in BD is solely based upon clinical phenomenology and individual interpretation. Thus, the neurobiological underpinnings of BD are of particular interest and under permanent investigation.

Emerging evidence suggests that immune-inflammatory activity and tryptophan catabolites (TRYCATs) changes are involved in the etiology as well as in the course of BD. TRYCATs are proposed to be mediators of immune-inflammatory activity and also neurodegeneration [7]. Depression and mania are accompanied by the activation of immune inflammatory pathways like increased levels of pro-inflammatory cytokines which are connected to alterations in the TRYCAT metabolism [8].

Tryptophan is an essential amino acid and precursor for two critical biochemical pathways relevant to the inflammatory neuropsychiatric interface involving the generation of neurotransmitter 5- hydroxytryptamine (5-HT, serotonin) and the formation of kynurenine (KYN) and its derivatives [9].

First interpretations of these pathways involved that pro-inflammatory conditions lead to an induction of indoleamine 2,3-dioxygenase-1 (IDO-1) under which tryptophan is primarily converted into KYN leading to reduced availability of tryptophan for serotonin biosynthesis and, consequently, altered serotonergic transmission in the brain with neuropsychiatric symptoms as a direct result of this cascade [10, 11]. However, as the brain may be able to compensate for the inflammation-induced decrease in circulating tryptophan, the serotonergic system may not be affected unfavorably as part of this process [11].

**Fig 1:**
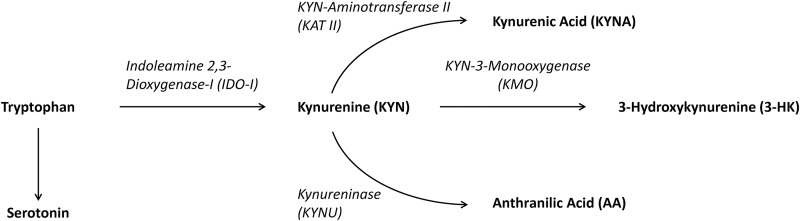
Kynurenine pathway. Enzymes are written in italic.

Recent research suggests that TRYCATs may exert effects independently of serotonin [11]. While KYN itself is inactive, it is further converted into different metabolites. KYN can be converted into three different branches (see Fig 1).

1. Due the enzymatic activity of kynurenine-3-monooxygenase (KMO), KYN is converted into 3-hydroxykynurenine (3-HK). Few enzymatic steps later in the cascade, 3-HK will be finally converted into quinolinic acid (QUIN). Both exert neurotoxic properties due to their ability to generate oxidative radicals and act as NMDA-receptor-agonists.
2. Due the enzymatic activity of KYN aminotransferase II (KATII), KYN is converted into kynurenic acid (KYNA), which as a NMDA-receptor-antagonist (like the antidementive drug memantine) and α7-nicotinic acetylcholine receptor-antagonist tends to be neuroprotective [11, 12]. Kynurenic acid also acts neuroprotective against excitotoxicity of QUIN, but an abnormal accumulation of KYNA beyond physiological levels could induce glutamatergic hypo-functioning and might disturb cognitive function [13]. High KYNA levels are also discussed to be involved in the pathophysiology of cognitive and psychotic features of schizophrenia [14].
3. KYN is a substrate for formation of anthranilic acid (AA), catalyzed by kynureninase (KYNU). Levels of AA were reported to be elevated in schizophrenia and the autoimmune diseases of rheumatoid arthritis and diabetes type 1 [15–17]. Less is reported about AA from a hypothetically relevant functional perspective. It might prevent tryptophan depletion, first by inhibiting sodium-transporters of uncharged solutes (e.g., tryptophan) across membranes [18], and second by serving as a substrate for bacteria metabolism, e.g. in the intestinal microbiome [19].

To date, studies on TRYCAT alterations in BD have only sporadically been conducted. Elevated levels of KYN and KYNA have been described in the post mortem anterior cingulate in BD patients with a history of psychosis [20]. CSF studies showed higher KYNA levels in 31 male BD patients compared to 23 male healthy controls (HC) [21], and notably being associated to manic and psychotic features [22, 23]. Johannson et al. [24] showed TRYCAT-alterations in ten BD patients (two of them were euthymic) with increases of 3-HK and KYNA synthesis in cultures of skin fibroblasts compared to twelve HC [24].

In the current investigation, we wanted to elucidate the peripheral measurements of KYN metabolism in patients with BD. To our knowledge there is no study investigating blood levels and ratios of key components of the KYN pathway in a large BD compared to a large HC sample. In clinical studies, evaluation of ratios between end-products and substrates are used for the estimation of activities of enzymes [25].

We therefore analyzed the 3-HK/KYN as a proxy of the KMO, KYNA/KYN as a proxy of KAT II and AA/KYN as a proxy of KYNU. We also evaluated the 3-HK/KYNA ratio, as this ratio is often used in literature as a proxy of neurotoxic vs. neuroprotective end-product relation [26]. To complete the analysis we also wanted to present the serum levels of the pathway (KYN, KYNA, 3-HK and AA) and see if they differ between BD and HC.

Our hypothesis was that euthymic BD show a bias to the more neurotoxic cascade of KYN derivatives compared to HC. In particular this would imply a relative increase of the conversion of KYN to the neurotoxic 3-HK in the BD sample, presuming an accelerated KMO activity and additionally we expected an increased 3-HK/KYNA ratio.

## Methods

The study was conducted at the Medical University of Graz, Department of Psychiatry. All patients took part in the ongoing single centre BIPFAT study, that assesses demographic parameters, complete actual and lifetime psychiatric history using the Structured Clinical Interview according to DSM-IV (SCID I), the psychiatric rating scales Hamilton-Depression (HAM-D) [27], Young Mania Rating Scale (YMRS) [28] and Beck’s Depression Inventory (BDI) [29], history of medication, anthropometric measure, blood pressure, fasting blood, cognitive testing, EEG, stool sample, different lifestyle questionnaires and magnetic resonance imaging (MRI) of the brain. All patients included were former in- or outpatients of the Medical University of Graz and had a diagnosis of BD I or BD II according to the DSM-IV criteria. Patients needed to be in the state of euthymia or mild depression (HAM-D score <14 and YMRS <9) and had given written informed consent prior to participating in the study.

The study has been approved by the local ethics committee (Medical University of Graz, Austria) in compliance with the current revision of the Declaration of Helsinki, ICH guideline for Good Clinical Practice and current regulations (EK-number: 24–123 ex 11/12).

Exclusion criteria were the presence of chronic obstructive pulmonary disease, rheumatoid arthritis, systemic lupus erythematosus, inflammatory bowel disease, neurodegenerative and neuroinflammatory disorders (i.e. Alzheimer's, Huntington's and Parkinson's disorder, multiple sclerosis), hemodialysis and interferon-α-based immunotherapy. Further exclusion criteria for controls were the presence of lifetime psychiatric diagnoses (verified by SCID I) and first and second grade relationship to relatives with psychiatric disorders. For further information about the study design and preliminary results see our previous reports [2, 30–33].

We took peripheral TRYCAT blood levels of 143 euthymic and mild depressive BD patients and 101 healthy controls. Targeted parameters were the levels of KYN, KYNA, 3-HK and AA, the ratio between end-products and substrates as proxies for the specific enzymatic activity (3-HK/KYN, KYNA/KYN, AA/KYN) as well as 3-HK/KYNA as a proxy for neurotoxic vs. neuroprotective end-product relation. Because of technical issues AA could only be evaluated in 113 BD and 88 HC.

For statistical analysis two multivariate analyses of covariance (MANCOVA) separated for targeted parameters involving AA (113 BD and 88 HC)and not involving AA (BD:143 and HC:101) controlled for age, sex, body mass index, presence of a cardiovascular disease and smoking were performed, as the groups differed in these parameters (see Table 1).

**Table 1:** Demographic and clinical parameters.

	BD (n = 143)	HC (n = 101)	*Statistics*	*p*
Male (%)*	55.9	39.6	***χ 2 = 6*.*323***	**.*012***
Age (years) (M, SD)*	43.9 (13.3)	40.3 (16.4)	***U = -2*.*392***	**.*017***
Body Mass Index (M, SD)**	28.2 (6.2)	24.7 (4.3)	***U = -4*.*954***	**.*000***
Cardiovascular Disease (%)*	29.4	26.8	***χ 2 = 5*.*035***	**.*025***
Smoking (%)**	48.3	26.7	***χ 2 = 10*.*502***	**.*001***
**Lithium (%)**	32.2	0	***n*.*a*.**	
**Antipsychotics (%)**	62.2	0	***n*.*a*.**	
**Antiepileptics (%)**	32.2	0	***n*.*a*.**	
**Two or more mood stabilizer (%)**	33.6	0	***n*.*a*.**	
**Antidepressant (%)**	68.5	0	***n*.*a*.**	
**Bipolar Disorder I (%)**	61.5	***n*.*a*.**	***n*.*a*.**	
**Bipolar Disorder II (%)**	35.0	***n*.*a*.**	***n*.*a*.**	

Note: Results from Chi square tests (*χ2*) and Mann-Whitney-U-tests (*U*). Statistically significant effects are marked bold. (n.a. = not applicable)

*p <.05,

** <.01

We didn’t control for medication because every BD patient had at least one kind of psychopharmacologic medication while HC didn’t. Thus it was simply not a feature applying to both groups and not suitable as a factor to control for. Moreover to address this point we analyzed if the targeted parameters differed in between patients with different kind of medications (lithium, antipsychotics, antiepileptics and antidepressants), which was not the case (data not shown).

### Biological assays/Quantification of KYN pathway metabolites (TRYCATs)

The chromatographic system was composed of a Waters Acquity UPLC separations module connected to a Xevo TQ MS triple-quadrupole mass spectrometer, equipped with a Z-spray ESI ion source (Waters Corp., Milford, MA, USA). Separation was carried out using a Kinetex XB-C18, 2.6 μm, 2.1 x 150 mm column (Phenomenex, Torrance, CA, USA).

Reagents for protein precipitation, derivatization, and chromatography were purchased from Sigma-Aldrich (St. Louis, MO, USA) and Biosolve (Valkenswaard, NL).

KYN, AA, 3-HK and KYNA, were purchased from Sigma-Aldrich. The internal standard KYNA-D5 was purchased from CDN Isotopes (Pointe-Claire, QC, Canada), KYN-D4 was purchased from Buchem BV (Minden, NL). Standards and a low and high quality control were established by adding defined amounts of each analyte to human serum samples (obtained from a blood bank). The human serum was necessary to take matrix effects into account, in order to cover concentrations below the analyte concentrations present in healthy humans the serum was diluted 1+1 with LC-MS/MS grade water and used as the lowest calibrator. The values of this calibrator were calculated by standard addition. As serum without these analytes is not available as there is no blank sample. A total volume of 300 μl serum samples, calibrators and controls was used for sample preparation. Analytes were extracted by adding 50 μl of 2.0 M urea and 50 μl of an internal standard solution containing KYN-D4 and KYNA-D5. Two precipitation steps by subsequently adding 200 μl methanol/ethanol (2/1 v:v) and 800 μl acetonitrile were carried out. The supernatant was separated into two portions, which were evaporated separately. One of these portions was directly reconstituted in mobile phase, while the other portion was derivatised with 200 μl HCl/Butanol at 90°C for 60 minutes. After evaporation this portion was reconstituted in mobile phase as well.

For chromatography, 7.5 μl of the reconstituted samples, calibrators and controls were loaded onto the LC-MS/MS system. The analytes were analysed in the underivatised sample. Gradient methods with a total duration of 7.5 min each were used for chromatographic separation. Mobile phase A was composed of 0.1% formic acid and 0.01% HFBA in water, mobile phase B was methanol. Flow rate was set at 0.25 ml/min, column temperature was set at 30.0°C. Retention times for the analytes were between 3.1 and 6.0 min.

The Xevo TQ MS was operating in atmospheric pressure and electrospray ionization in positive mode (ESI+). Ion source settings were: capillary voltage, 1.00 kV; desolvation temperature 650°C; source temperature, 150°C; nitrogen was used as desolvation gas with an API gas flow rate of 1200 l/h; argon was used as collision gas at a flow rate of 0.15 ml/min. The analytes and internal standards were detected using multi reaction monitoring (MRM) technique. System operation, data acquisition and data processing were controlled using MassLynx V4.1 software (Waters Corp.). The Lower Limit of Quantification and Lower Limit of Detection of the used method were calculated according to DIN 32645 guidelines. The method was further validated based on the EMEA guidelines at the Institute of Laboratory Medicine, Medical Center of Ludwig Maximilian University, Munich, Germany.

## Results

**Fig 2:**
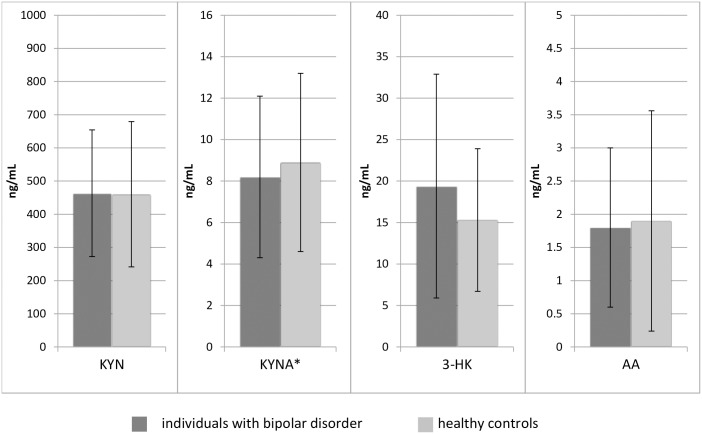
Serum levels of TRYCATs in ng/mL. Results from MANCOVA controlled for age, sex, body mass index, presence of a cardiovascular disease and smoking; *p<0.05; KYN = kynurenine, KYNA = kynurenic acid, 3-HK = 3-hydroxykynurenine, AA = anthranilic acid.

**Fig 3:**
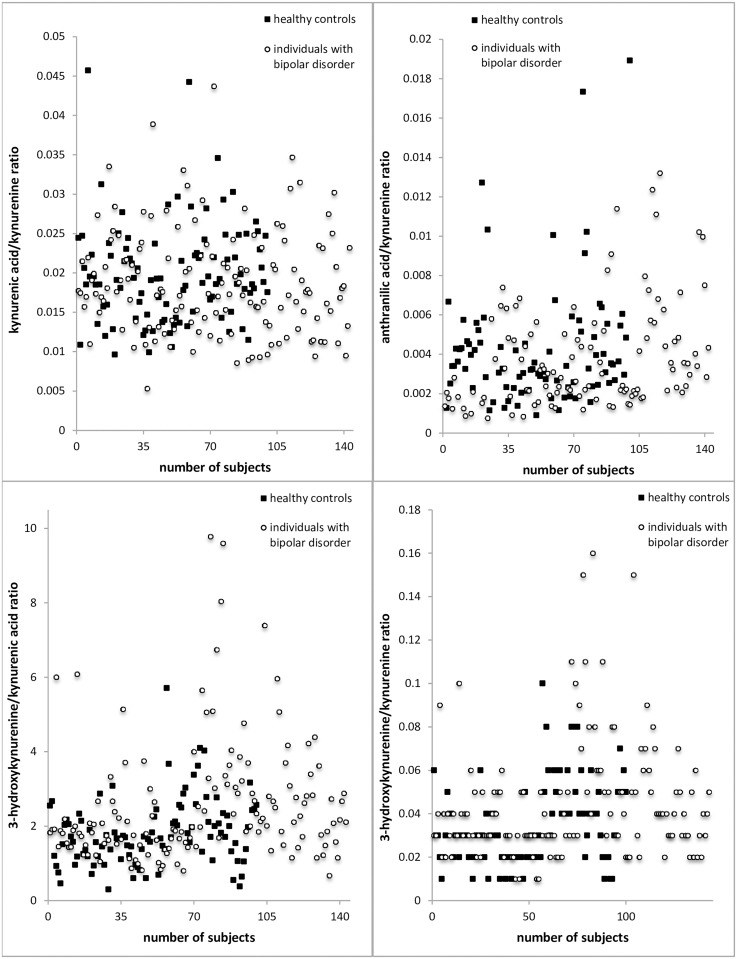
Scatter graphs showing the distribution of the serum levels of the measured TRYCATs in ng/mL.

The levels of KYNA (*F = 5*,*579; p <*.*05*) were reduced in BD compared to HC (see Figs 2 and 3). There was no statistical difference in the other parameters.

**Fig 4:**
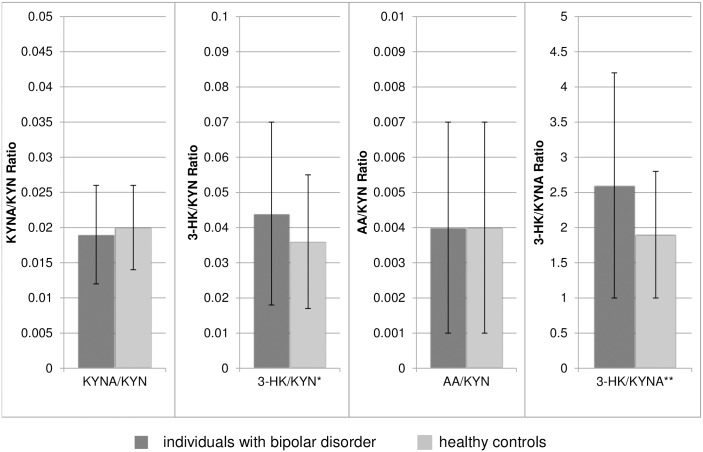
Ratios of TRYCAT metabolism. Results from MANCOVA controlled for age, sex, body mass index, presence of a cardiovascular disease and smoking; *p<0.05, **p<0.01; KYN = kynurenine, KYNA = kynurenic acid, 3-HK = 3-hydroxykynurenine, AA = anthranilic acid.

**Fig 5:**
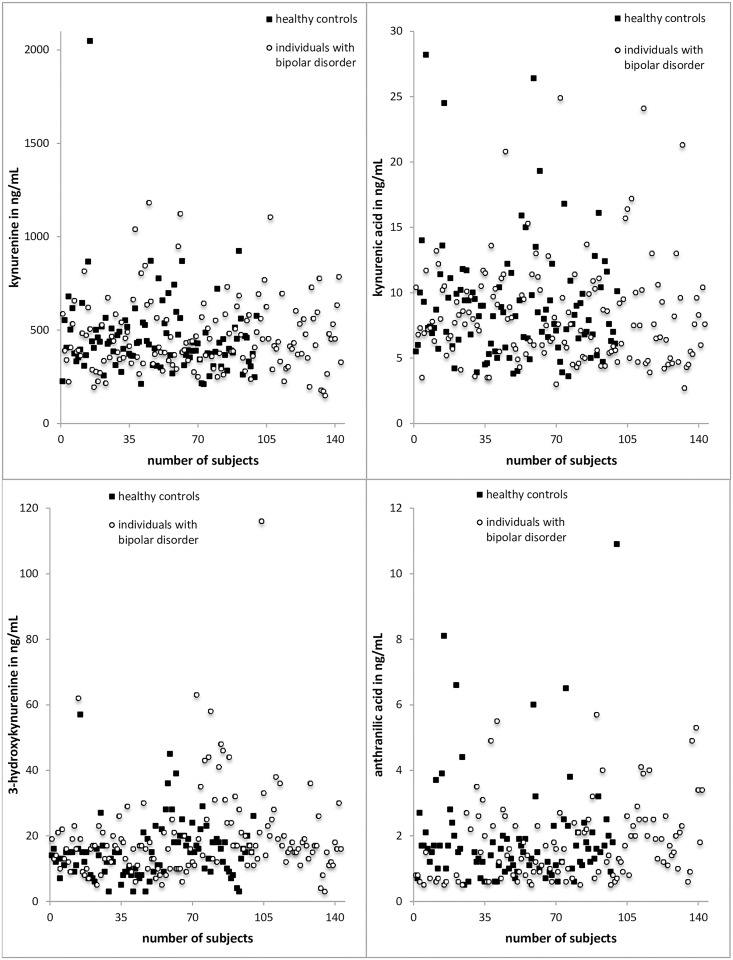
Scatter graphs showing the distributions of the TRYCAT metabolism ratios.

The approximated enzymatic activity of the kynurenine-3-monooxygenase (KMO) reflected by the 3-HK/KYN ratio was increased in BD individuals compared to HC (*F = 5*,*394; p <*.*05*). Additionally the ratio of 3-HK/KYNA was increased in individuals with BD compared to HC (*F = 11*,*357; p <*.*01*) (see Figs 4 and 5). There was no statistical difference in the other investigated ratios.

Chromatograms of the investigated tryptophan pathway intermediates analyzed with high pressure liquid chromatography coupled with mass spectrometry (HPLC-MS/MS) in Multiple Reaction Monitoring (MRM) are shown in the supplementary S1 File.

## Discussion

The aim of the present study was to elucidate the KYN pathway in euthymic individuals with BD. We wanted to evaluate the serum levels of KYN, KYNA, 3-HK and AA as well as the approximated activity of the KMO (measured by 3-HK/KYN ratio), KAT II (measured by KYNA/KYN ratio), KYNU (measured by AA/KYN ratio) and the relation of the neurotoxic TRYCAT 3-HK compared to the more neuroprotective TRYCAT KYNA (measured by 3-HK/KYNA ratio) between euthymic individuals with BD and HC.

The results presented here give further supporting evidence for the contribution of TRYCATs, in particular KYN and its derivatives, to the pathophysiology of BD.

We could show a significant bias to the neurotoxic branch of KYN breakdown. The level of KYNA was significantly decreased in individuals with BD, while the increase in 3-HK levels compared to HCs didn’t reach statistical significance. There was a significant increase of 3-HK compared to the more neuroprotective KYNA, evident by an increased 3-HK/KYNA ratio, as well as an increased 3-HK/KYN ratio, as a proxy for KMO activity, in a sample of 143 euthymic BD patients compared to 101 HC. There was no significant difference of the approximated KAT II or KYNU activity between BD and HC.

TRYCATs are proposed to be mediators of immune-inflammatory activity and also neurodegeneration [7]. This increase of the neurotoxic catabolite 3-HK compared to the neuroprotective KYNA even in the state of euthymia might relate to cognitive dysfunction, which is prevalent in acute phases of BD, but also frequently prevails in euthymia [1, 2]. These processes might also be partially responsible for unspecific brain structural changes in BD, like reduction in grey and white matter, increased white matter lesions and microstructural changes which can be found in various Magnetic resonance imaging (MRI)-studies [3–6]. Alterations in different immune-inflammatory processes are evident in BD. In particular its implications to the KYN pathway have been highlighted by the study of Johansson et al. [24], which showed that pro-inflammatory cytokine treatment (with human recombinants of interleukin-1, interleukin-6, tumor-necrosis-factor and interferon-α (IFN-α) led to an IDO-1 activation and further increase of the already elevated 3-HK/KYNA ratio, in cultures of skin fibroblasts, suggesting that pro-inflammatory states are linked to an even greater imbalance. Raisson et al. [34] showed a significant increase towards the KYN-pathway metabolism combined with associated increase of depressive symptoms in 27 hepatitis C following a IFN-α treatment. The IDO-1 activity in general is enhanced by inflammatory pathways. We could show this in our previous preliminary results on increased IDO-1 activity in euthymic BD patients compared to HC with greater increases noted in the subsample of overweight BD patients [30]. However, the tryptophan breakdown in peripheral blood of individuals with BD has not been extensively studied to date.

Accumulating evidence suggests that mild inflammatory processes in the periphery and the brain are involved in the pathophysiology of BD across manic and depressive episodes but may prevail throughout the euthymic “between-episode” period as a chronic process [35, 36]. The euthymia in BD is of special interest, as it is characterized by the absence of affective symptoms and the confounding of a state effect is removed. We also controlled for the potential confounding factors obesity, smoking and cardiovascular disease, as they are all linked to low grade inflammation [37–39] and were significantly more common in the investigated BD group in our cohort, as expected due different earlier studies [40–42].

As a result of these foregoing observations it seems like we could at least hypothetically think about crafting an approach to a possible biomarker (or at least a part of a combined biomarker with other parameters) for the recognition and staging of disease episodes in BD. As manic and depressive episodes seem to be accompanied by increased inflammation [35, 36], a measureable individual activation of the KYN-pathway could be detectable. To further elucidate that process a longitudinal assessment-approach across episodes in individuals with BD seems like a good way to achieve that information.

### Limitations

The mismatch of BD patients and healthy controls in important parameters, even if we controlled for their influence, especially the difference in age is a limiting factor of the study. The medication might also be of limiting contribution, as all BD individuals had psychopharmacological treatment, while no HC had these kinds of medications. To address this point we analyzed if the targeted parameters differed in between patients with different kind of medications. Despite this was not the case in our sample, the influence of ongoing drug treatment among patients can still not be ruled out. The study is also limited by its cross-sectional design. The contribution of neurotoxic TRYCATs to cognitive decline and brain structural abnormalities despite being logically sound is only hypothetical. Another limitation comes from the fact that we took peripheral levels of the targeted parameters. This could be a problem for the interpretation of KYNA levels and ratios as animal models teach us that KYNA diffuse only in very low rate beyond the brain-blood barrier [43]. However, other authors propose that the same processes involving KYN catabolism in the brain should basically also take place in the periphery [8, 34]. The other TRYCATS (KYN, 3-HK and AA) are proposed to pass the brain-blood barrier easily [43, 44]. Nevertheless, as we are unable to precisely relate the peripheral measurement of the investigated metabolites to the central nervous system in general and especially not to the relevant brain regions or circuits, it seems far too speculative to say these markers should be seen in a causative role for the disorder itself. In this context, we have to point out that the investigated plasma metabolites should rather be seen as surrogate markers.

A further limitation is the use of the 3-HK/KYN ratio as a proxy of the KMO activity as it may also reflect increased availability of precursor, i.e. kynurenine, especially in the view of decreased conversion of KYN to KYNA. Considering the fact that the enzyme does not operate under full saturation, bigger pool of KYN will be also be converted into 3-HK without noticeable changes in free KYN levels.

### Conclusion and future directions

In conclusion our findings subserve the concept of KYN-pathway alterations in the pathophysiology of BD. We present evidence of increased breakdown towards the neurotoxic branch in KYN metabolism, which could be involved in the development of brain structural changes and cognitive deficits in BD although direct extrapolation from observations made in serum to brain cell metabolism remains elusive. Further research should focus on state dependent changes in TRYCATs and its relation to cognition, brain structure and staging parameters favorably in a longitudinal design across different episodes of disease. Moreover, the contribution of sex and metabolic parameters seems to be of special interest and should be further analyzed.

## Supporting information

S1 File Chromatograms of tryptophan pathway intermediates. Chromatograms of the investigated tryptophan pathway intermediates analyzed with high pressure liquid chromatography coupled with mass spectrometry (HPLC-MS/MS) in Multiple Reaction Monitoring (MRM).
(PDF)

## References

[1] Robinson LJ, Ferrier IN. (2006) Evolution of cognitive impairment in bipolar disorder: A systematic review of cross-sectional evidence. Bipolar Disord 8(2): 103–116. 10.1111/j.1399-5618.2006.00277.x 16542180

[2] Lackner N, Bengesser SA, Birner A, Painold A, Fellendorf FT, Platzer M et al (2015) Abdominal obesity is associated with impaired cognitive function in euthymic bipolar individuals. World J Biol Psychiatry: 1–12. 10.3109/15622975.2015.1046917 26068130

[3] Arnone D, Cavanagh J, Gerber D, Lawrie SM, Ebmeier KP, McIntosh AM. (2009) Magnetic resonance imaging studies in bipolar disorder and schizophrenia: Meta-analysis. Br J Psychiatry 195(3): 194–201. 10.1192/bjp.bp.108.059717 19721106

[4] Kempton MJ, Geddes JR, Ettinger U, Williams SC, Grasby PM. (2008) Meta-analysis, database, and meta-regression of 98 structural imaging studies in bipolar disorder. Arch Gen Psychiatry 65(9): 1017–1032. 10.1001/archpsyc.65.9.1017 18762588

[5] Beyer JL, Young R, Kuchibhatla M, Krishnan KR. (2009) Hyperintense MRI lesions in bipolar disorder: A meta-analysis and review. Int Rev Psychiatry 21(4): 394–409. 10.1080/09540260902962198 20374153 PMC4098150

[6] Nortje G, Stein DJ, Radua J, Mataix-Cols D, Horn N. (2013) Systematic review and voxel-based meta-analysis of diffusion tensor imaging studies in bipolar disorder. J Affect Disord 150(2): 192–200. 10.1016/j.jad.2013.05.034 23810479

[7] Anderson G, Maes M. (2013) Metabolic syndrome, alzheimer disease, schizophrenia, and depression: Role for leptin, melatonin, kynurenine pathways, and neuropeptides In: Faroqui T F A, editor. Metabolic Syndome and neurolgical disorders.: Wiley pp. 235–248.

[8] Anderson G, Maes M. (2015) Bipolar disorder: Role of immune-inflammatory cytokines, oxidative and nitrosative stress and tryptophan catabolites. Curr Psychiatry Rep 17(2): 8-014-0541-1. 10.1007/s11920-014-0541-1 25620790

[9] Wirleitner B, Neurauter G, Schrocksnadel K, Frick B, Fuchs D. (2003) Interferon-gamma-induced conversion of tryptophan: Immunologic and neuropsychiatric aspects. Curr Med Chem 10(16): 1581–1591. 12871129 10.2174/0929867033457179

[10] Widner B, Laich A, Sperner-Unterweger B, Ledochowski M, Fuchs D. (2002) Neopterin production, tryptophan degradation, and mental depression—what is the link? Brain Behav Immun 16(5): 590–595. 12401473 10.1016/s0889-1591(02)00006-5

[11] Dantzer R, O'Connor JC, Lawson MA, Kelley KW. (2011) Inflammation-associated depression: From serotonin to kynurenine. Psychoneuroendocrinology 36(3): 426–436. 10.1016/j.psyneuen.2010.09.012 21041030 PMC3053088

[12] Schwarcz R, Bruno JP, Muchowski PJ, Wu HQ. (2012) Kynurenines in the mammalian brain: When physiology meets pathology. Nat Rev Neurosci 13(7): 465–477. 10.1038/nrn3257 22678511 PMC3681811

[13] Myint AM. (2012) Kynurenines: From the perspective of major psychiatric disorders. Febs j 279(8): 1375–1385. 10.1111/j.1742-4658.2012.08551.x 22404766

[14] Erhardt S, Schwieler L, Imbeault S, Engberg G. (2016) The kynurenine pathway in schizophrenia and bipolar disorder. Neuropharmacology. 10.1016/j.neuropharm.2016.05.020 27245499

[15] Oxenkrug G, van der Hart M, Summergrad P. (2015) Elevated anthranilic acid plasma concentrations in type 1 but not type 2 diabetes mellitus. Integr Mol Med 2(5): 365–368. 10.15761/IMM.1000169 26523229 PMC4624227

[16] Oxenkrug G, van der Hart M, Roeser J, Summergrad P. (2016) Anthranilic acid: A potential biomarker and treatment target for schizophrenia. Ann Psychiatry Ment Health 4(2): 1059 Epub 2016 Jan 30. 27042691 PMC4817843

[17] Igari T, Tsuchizawa M, Shimamura T. (1987) Alteration of tryptophan metabolism in the synovial fluid of patients with rheumatoid arthritis and osteoarthritis. Tohoku J Exp Med 153(2): 79–86. 5349889 10.1620/tjem.99.73

[18] Pajor AM. (2006) Molecular properties of the SLC13 family of dicarboxylate and sulfate transporters. Pflugers Arch 451(5): 597–605. 10.1007/s00424-005-1487-2 16211368 PMC1866268

[19] Rydon HN. (1948) Anthranilic acid as an intermediate in the biosynthesis of tryptophan by bact. typhosum. Br J Exp Pathol 29(1): 48–57. 18865104 PMC2073081

[20] Miller CL, Llenos IC, Dulay JR, Weis S. (2006) Upregulation of the initiating step of the kynurenine pathway in postmortem anterior cingulate cortex from individuals with schizophrenia and bipolar disorder. Brain Res 1073–1074: 25–37. 10.1016/j.brainres.2005.12.056 16448631

[21] Olsson SK, Samuelsson M, Saetre P, Lindstrom L, Jonsson EG, Nordin C et al (2010) Elevated levels of kynurenic acid in the cerebrospinal fluid of patients with bipolar disorder. J Psychiatry Neurosci 35(3): 195–199. 20420770 10.1503/jpn.090180 PMC2861136

[22] Lavebratt C, Olsson S, Backlund L, Frisen L, Sellgren C, Priebe L et al (2014) The KMO allele encoding Arg452 is associated with psychotic features in bipolar disorder type 1, and with increased CSF KYNA level and reduced KMO expression. Mol Psychiatry 19(3): 334–341. 10.1038/mp.2013.11 23459468 PMC4990004

[23] Olsson SK, Sellgren C, Engberg G, Landen M, Erhardt S. (2012) Cerebrospinal fluid kynurenic acid is associated with manic and psychotic features in patients with bipolar I disorder. Bipolar Disord 14(7): 719–726. 10.1111/bdi.12009 23030601

[24] Johansson AS, Owe-Larsson B, Asp L, Kocki T, Adler M, Jerker H et al (2013) Activation of kynurenine pathway in ex vivo fibroblasts from patients with bipolar disorder or schizophrenia: Cytokine challenge increases production of 3-hydroxykynurenine. J Psychiatr Res 47(11): 1815–1823. 10.1016/j.jpsychires.2013.08.008 24012176

[25] Ulvik A, Theofylaktopoulou D, Midttun O, Nygard O, Eussen SJ, Ueland PM. (2013) Substrate product ratios of enzymes in the kynurenine pathway measured in plasma as indicators of functional vitamin B-6 status. Am J Clin Nutr 98(4): 934–940. 10.3945/ajcn.113.064998 24004893

[26] Lovelace MD, Varney B, Sundaram G, Lennon MJ, Lim CK, Jacobs K et al (2016) Recent evidence for an expanded role of the kynurenine pathway of tryptophan metabolism in neurological diseases. Neuropharmacology. 10.1016/j.neuropharm.2016.03.024 26995730

[27] Hamilton M, White J. (1960) A rating scale for depression. Journal of Neurology, Neurosurgery, and Psychiatry (23): 56–62. 14399272 10.1136/jnnp.23.1.56 PMC495331

[28] Young RC, Biggs JT, Ziegler VE, Meyer DA. (1978) A rating scale for mania: Reliability, validity and sensitivity. Br J Psychiatry 133: 429–435. 728692 10.1192/bjp.133.5.429

[29] Beck AT, Steer RA, Hautzinger M. (1994) Beck-Depressions-Inventar:(BDI).

[30] Reininghaus EZ, McIntyre RS, Reininghaus B, Geisler S, Bengesser SA, Lackner N et al (2014) Tryptophan breakdown is increased in euthymic overweight individuals with bipolar disorder: A preliminary report. Bipolar Disord 16(4): 432–440. 10.1111/bdi.12166 24330408

[31] Reininghaus EZ, Lackner N, Fellendorf FT, Bengesser S, Birner A, Reininghaus B et al (2015) Weight cycling in bipolar disorder. J Affect Disord 171: 33–38. 10.1016/j.jad.2014.09.006 25443762

[32] Bengesser SA, Lackner N, Birner A, Fellendorf FT, Platzer M, Mitteregger A et al (2015) Peripheral markers of oxidative stress and antioxidative defense in euthymia of bipolar disorder-gender and obesity effects. J Affect Disord 172: 367–374. 10.1016/j.jad.2014.10.014 25451439

[33] Lackner N, Mangge H, Reininghaus EZ, McIntyre RS, Bengesser SA, Birner A et al (2015) Body fat distribution and associations with metabolic and clinical characteristics in bipolar individuals. Eur Arch Psychiatry Clin Neurosci 265(4): 313–319. 10.1007/s00406-014-0559-8 25381166

[34] Raison CL, Dantzer R, Kelley KW, Lawson MA, Woolwine BJ, Vogt G et al (2010) CSF concentrations of brain tryptophan and kynurenines during immune stimulation with IFN-alpha: Relationship to CNS immune responses and depression. Mol Psychiatry 15(4): 393–403. 10.1038/mp.2009.116 19918244 PMC2844942

[35] Kim YK, Jung HG, Myint AM, Kim H, Park SH. (2007) Imbalance between pro-inflammatory and anti-inflammatory cytokines in bipolar disorder. J Affect Disord 104(1–3): 91–95. 10.1016/j.jad.2007.02.018 17434599

[36] Langan C, McDonald C. (2009) Neurobiological trait abnormalities in bipolar disorder. Mol Psychiatry 14(9): 833–846. 10.1038/mp.2009.39 19455151

[37] Danesh J, Whincup P, Walker M, Lennon L, Thomson A, Appleby P et al (2000) Low grade inflammation and coronary heart disease: Prospective study and updated meta-analyses. Bmj 321(7255): 199–204. 10903648 10.1136/bmj.321.7255.199 PMC27435

[38] Yanbaeva DG, Dentener MA, Creutzberg EC, Wesseling G, Wouters EF. (2007) Systemic effects of smoking. Chest 131(5): 1557–1566. 10.1378/chest.06-2179 17494805

[39] Nishimura S, Manabe I, Nagai R. (2009) Adipose tissue inflammation in obesity and metabolic syndrome. Discov Med 8(41): 55–60. 19788868

[40] Goldstein BI, Fagiolini A, Houck P, Kupfer DJ. (2009) Cardiovascular disease and hypertension among adults with bipolar I disorder in the united states. Bipolar Disord 11(6): 657–662. 10.1111/j.1399-5618.2009.00735.x 19689508 PMC3401900

[41] Diaz FJ, James D, Botts S, Maw L, Susce MT, De Leon J. (2009) Tobacco smoking behaviors in bipolar disorder: A comparison of the general population, schizophrenia, and major depression. Bipolar Disord 11(2): 154–165. 10.1111/j.1399-5618.2009.00664.x 19267698

[42] McIntyre RS, Danilewitz M, Liauw SS, Kemp DE, Nguyen HT, Kahn LS, et al (2010) Bipolar disorder and metabolic syndrome: An international perspective. J Affect Disord 126(3): 366–387. 10.1016/j.jad.2010.04.012 20541810

[43] Fukui S, Schwarcz R, Rapoport SI, Takada Y, Smith QR. (1991) Blood-brain barrier transport of kynurenines: Implications for brain synthesis and metabolism. J Neurochem 56(6): 2007–2017. 1827495 10.1111/j.1471-4159.1991.tb03460.x

[44] Campbell B, Pocivavcek A, Notarangelo F, Parachikova A. (2015) The role of kynurenine pathway metabolites in neuropsychiatric disorders In: Mittal Sandeep, editor. Targeting the Broadly Pathogenic Kynurenine Pathway.: Springer pp. 241–254.

