# Supplementary material for: Increased breakdown of kynurenine towards its neurotoxic branch in bipolar disorder
Source: PLoS One. 2017 Feb 27;12(2):e0172699. doi: 10.1371/journal.pone.0172699 (PMC5328271; doi:10.1371/journal.pone.0172699)
Supplement: S1 File — Chromatograms of the investigated tryptophan pathway intermediates analyzed with high pressure liquid chromatography coupled with mass spectrometry (HPLC-MS/MS) in Multiple Reaction Monitoring (MRM). (PDF) [file pone.0172699.s001.pdf]

Analytes measured underivatised

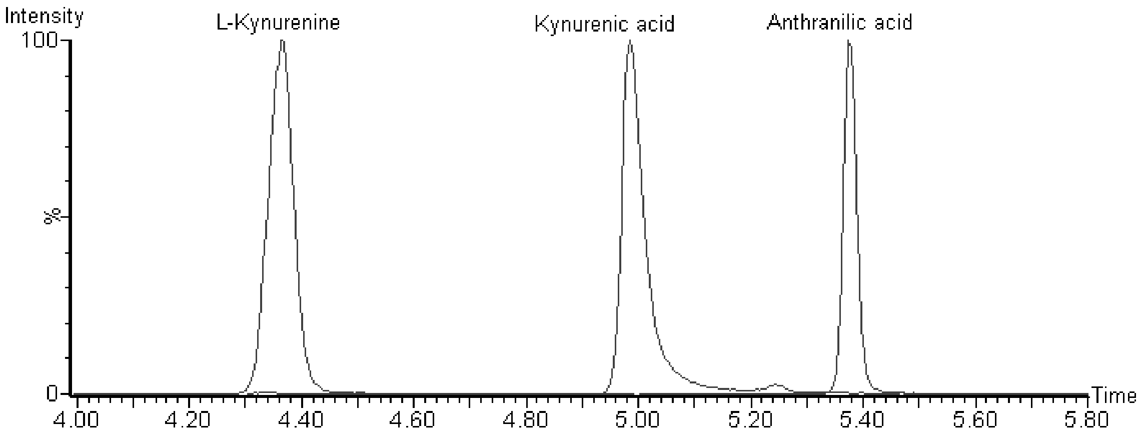

L-Kynurenine

RT: 4.36 min
MRM 1: m/z 209.1 > 192.1
MRM 2: m/z 209.1 > 94.1

Kynurenic acid

RT: 4.99 min
MRM 1: m/z 190.1 > 144.1
MRM 2: m/z 190.1 > 116.1

Anthranilic acid

RT: 5.37 min
MRM 1: m/z 138.1 > 120.1
MRM 2: m/z 138.1 > 92.1

Analyte measured derivatised
(separate HPLC-MS/MS measurement)

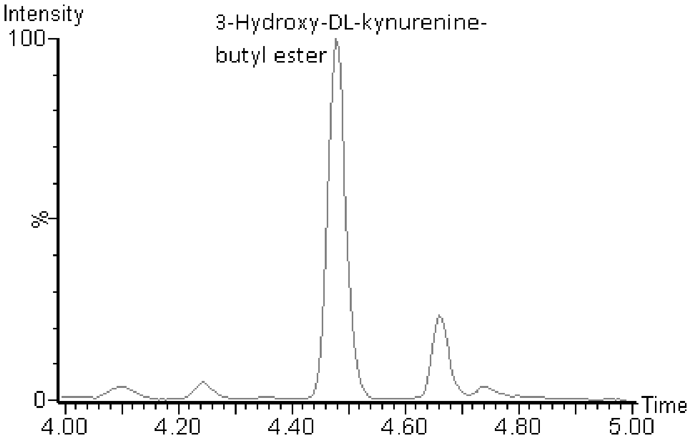

3-Hydroxy-DL-kynurenine -butyl ester

RT: 4.48 min
MRM 1: m/z 281.2 > 264.1
MRM 2: m/z 281.2 > 152.1

Abbreviations:
RT = Retention Time
MRM = Multiple Reaction Monitoring

Legend:
Chromatograms of tryptophan pathway intermediates analysed with high pressure liquid chromatography coupled with mass spectrometry (HPLC-MS/MS) in Multiple Reaction Monitoring (MRM)
